# Investigating the Role of Silica in Thermo-Oxidative Degradation of EPDM Recycled Composites for Applications in Building and Construction

**DOI:** 10.3390/polym18020250

**Published:** 2026-01-16

**Authors:** Xavier Colom, Leire Moral, Javier Cañavate

**Affiliations:** Department of Chemical Engineering, ESEIAAT-UPC, Colom, 1, 08222 Terrassa, Spain; leire.moral@estudiantat.upc.edu (L.M.); francisco.javier.canavate@upc.edu (J.C.)

**Keywords:** EPDM, construction and building, thermooxidation, acoustic behavior, elastomeric compounds

## Abstract

This work investigates the structural, acoustic, and thermo-oxidative degradation behavior of elastomeric composites made from neat EPDM and recycled devulcanized EPDM (EPDMd) blends, both with and without silica (SiO_2_). SiO_2_ plays a complex role in degradation, possibly acting as a catalyst and also affecting the properties of the materials. Samples were subjected to accelerated degradation at 80 °C for 30 days. The characterization included the mechanical, spectroscopical (FTIR-ATR), thermal (TGA), and morphological (SEM) studies of the samples. Given EPDM’s use in construction as a sound-absorber, its acoustic properties were also analyzed. The determination of the mechanical properties shows that the incorporation of SiO_2_ improves the Young’s modulus (YM), maintains the tensile strength (TS) at similar values, and causes a decrease in elongation at break (EB). The content of EPDMd slightly decreases both the TS and the EB and increases the YM. The thermo-oxidative degradation of the studied composites does not affect the TS values, but it increases the YM for the samples with and without SiO_2_ for EPDMd contents higher than 40 phr, and decreases the EB for samples with and without SiO_2_ for all EPDMd contents. The FTIR-ATR, TGA, and SEM results show that the addition of SiO_2_ catalyzes the thermo-oxidative degradation process, while the EPDMd inhibits structural degradation. Migration of the ZnSt_2_ included in the formulations to the surface is common in these elastomers. In this case, EPDMd forms microaggregates, which retain the exudation of ZnSt_2_ crystals, especially in the non-degraded samples. The degraded samples present irregular structures, with microcavities, cracks, and occlusions, which increase the acoustic absorption mainly at frequencies below 1500 Hz.

## 1. Introduction

The urgent global focus on sustainability requires considerable effort to address critical issues related to materials, including pollution, waste generation, atmospheric emissions, and overall environmental impact. The outcomes of major international forums, such as COP30 in Belém (2025), underscore the commitment to combat climate change through actions like reducing greenhouse gas emissions, mobilizing climate adaptation funds, and strengthening international cooperation [1]. This global mandate to reduce reliance on fossil fuels has a significant, direct impact on materials derived from these resources, including synthetic rubbers such as EPDM (ethylene–propylene–diene monomer). EPDM is an important elastomeric material that will be affected by mandates to transition away from fossil feedstocks. Consequently, there is an imperative need to implement strategies to reduce its virgin consumption and maximize the reuse and high-value recovery of the significant EPDM waste generated annually, driving research toward effective recycling and material circularity solutions.

EPDM (ethylene–propylene–diene monomer) is situated among the most widely used elastomers, particularly in civil engineering, and has experienced substantial growth in use over the last decade [2,3]. EPDM’s versatility and strong market presence stem from its combination of properties, making it highly valuable in the construction, building, and automotive sectors. These beneficial characteristics include (i) superior resistance to weathering and UV radiation, ensuring durability in outdoor conditions; (ii) excellent low-temperature flexibility, making it ideal for seals and gaskets in cold climates; (iii) good heat resistance, crucial for high-temperature applications like vehicle engines; (iv) chemical inertness to a wide variety of acids, alkalis, and solvents; (v) excellent sealing ability and low water vapor permeability, offering effective moisture barriers for roofing and window construction; and (vi) inherent resistance to wear and long-term aging [4,5]. Because of this advantageous property profile, EPDM has become a ubiquitous material, ranging from seals and gaskets in the automotive industry to critical waterproofing membranes, acoustic panels, and insulation in construction, wherever weather resistance is critical. Furthermore, its capacity for energy dissipation makes it useful for damping elements within the building industry [6,7,8].

The necessity of mitigating the significant volume of elastomeric waste generated annually across various societal sectors necessitates the development of new, efficient recycling processes. The difficulty in the reuse of these materials lies in the crosslinking structure, and also in the contamination when being in contact with other materials like cement, etc. The recycling of industrial waste coming from its own manufacturing process, where the main material is clean, is easier to recover [9,10].

In order to avoid the limitations imposed by the crosslinked structure, Colom et al. have reported that a thermo-mechanical and microwave combined devulcanization process produced an intense devulcanization effect [11] with a significant cleavage of the crosslinking chains. The presence of BPO has an important influence on devulcanization, and there is a relation between the effect of the temperature and the amount of BPO [12]. Sousa et al. have analyzed the devulcanization of waste EPDM using the microwave process. According to their findings, the microwave process is very efficient because it causes breaking mainly in the S-S crosslinks, respecting the main macromolecular chains [13,14], but has the inconvenience of forming secondary cross-links [15]. In order to improve the degree of devulcanization and to minimize the formation of these secondary crosslinks, combined thermomechanical and microwave processes have been proposed, which combine mechanical shear and controlled heating to selectively break the sulfur crosslinks while preserving, as much as possible, the main polymer backbone. This approach allows for a more homogeneous devulcanization and better recovery of the elastomeric properties of the recycled material [16,17]. The devulcanized EPDM can be used in blends with neat EPDM in order to obtain useful materials.

In application, EPDM, whether in natural environments typical of the building sector or industrial processes, is continually subjected to various degradation conditions, primarily involving exposure to relatively high temperatures and environmental factors. Despite the need for ways to recycle EPDM and the promising possibilities of using EPDM–EPDMd (devulcanized EPDM) blends, the long-term stability and functional performance of these recycled composites remain underexplored.

SiO_2_ is a common filler used in EPDM and other elastomers. Its role in the properties of complex materials, including devulcanized EPDM, has not been extensively studied. Furthermore, its influence on the degradation process is not yet well understood. Basically, the silica is suspected to catalyze degradation by several mechanisms related to the changes induced in the polymer chain dynamics or by its effect in facilitating the action of oxidative agents. Several mechanisms will be further commented on when discussing the results.

This work is therefore dedicated to investigating the behavior of EPDM–EPDMd blends with and without SiO_2_ subjected to an accelerated thermo-oxidative degradation process (80 °C in a convection oven for 30 days). The core focus is to research the effect of degradation and determine the precise influence of the SiO_2_ reinforcing agent on the degradation mechanism and the resulting structural evolution. Furthermore, given the widespread use of EPDM in civil applications as acoustic insulators, the changes in the acoustic absorption properties of these composites following degradation are also analyzed. This study aims to establish a link between composition, degradation, and functional performance in sustainable EPDM composites.

## 2. Materials and Methods

### 2.1. Materials

Waste EPDM from commercial, industrial, and residential roofing origins was supplied by Firestone Building Products Terrassa (Terrassa, Spain). According to the producer, the material had a composition of 100 phr EPDM, 15 phr aluminum silicate, 35 phr Carbon Black, 15 phr non-polar plasticizer, and 2 phr of the curing system based on ZnO, N-cyclohexyl-2-benzothiazolesulphenamide (CBS), and sulfur.

Neat EPDM, benzoyl peroxide (BPO), carbon black N-330 (CB), vulcanization accelerators (TBBS-N-tert-butyl-2-benzothiazole sulphenamide, TMTD—tetramethylthiuram disulfide), CBS, stearic acid (StA), zinc oxide (ZnO), and sulfur (S) of technical purity were supplied by Vigar (Vilafranca del Panadès, Spain). StA and ZnO act as activators, facilitating and improving the vulcanization process.

Amorphous silica (synthetic amorphous silica—SAS) was obtained by chemical synthesis (acid precipitation). Micro/nanoparticles of SiO_2_ (83%): the particles have a size below 150 µm and a specific surface area of 180 m^2^/g, indicating a very fine morphology.

### 2.2. Devulcanization

Combined thermo-mechanical and microwave devulcanization processes have been performed in two steps: thermo-mechanical treatment in Brabender plastograph equipment (München, Germany) and microwave irradiation in a prototype microwave oven (Terrassa, Spain) adapted with a motorized stirring system made of PTFE according to a report in a previous manuscript [11].

### 2.3. Sample Preparation

EPDMd/EPDM compounds were prepared at 70 °C using a Brabender plastograph batch mixer. The rotational speed of the rollers was 100 rpm. The mixing time was 8 min, which included 2 min of preliminary mastication of neat EPDM, 4 min of mixing with devulcanized EPDM (0, 10, 20, 40, and 50 parts per hundred of rubber (phr), 30 phr of carbon black, and 15 phr of SiO_2_), and 2 min of mixing the blend with the sulfur curing system. The same curing system was used for all samples. The curing system comprised (phr) zinc oxide 5.0, stearic acid 3.0, TBBS 1.0, TMTD 0.25, and sulfur 2.0.

After using the Brabender, the granulate mixture obtained was taken into a two-roll mill to form a thin film that would later be placed into a mold. The two-roll mill works at room temperature.

The obtained EPDM/EPDMd samples were molded into 3 mm thick samples at 160 °C for 12 min under a pressure of 4.9 MPa using a laboratory plate press type P 200E from Dr. Collin GmbH (Ebersberg, Germany). The samples were coded as EPDMXEPDMd SiO_2_, where X means the amount of EPDMd and SiO_2_ means that the samples either incorporate SiO_2_ or do not.

### 2.4. Thermo-Degradation Process

EPDM samples were subjected to thermal aging in a ventilated oven at 80 °C (J.P. Selecta S.A. Abrera, Spain) in ambient air conditions to evaluate their thermo-oxidative stability. The specimens were placed on metal trays to ensure uniform heat exposure and were aged for 30 days. At these intervals, the samples were removed, cooled to room temperature, and characterized to determine changes in mass, mechanical properties, and chemical structure. The process simulates long-term service conditions by promoting gradual oxidation, post-curing, and chain-scission reactions, allowing the assessment of the material’s resistance to thermal degradation.

### 2.5. Rationale for the Selected Aging Protocols for Building and Construction Materials

The methodologies employed in this study are directly related to real-world building-exposure scenarios. Thermo-oxidative aging (30 days at 80 °C) was conducted to simulate years of accelerated degradation for rooftop or facade components exposed to the sun. Since polymeric materials in construction undergo chain scission via thermal oxidation, this study evaluates whether the inclusion of recycled EPDM (EPDMd) and silica fillers accelerates or mitigates this degradation. Abrasion and hardness testing are critical for materials that form part of pavements or dynamic joints that must withstand physical wear during the useful life of the building.

### 2.6. Measurements

TGA was performed on a Perkin Elmer TGA 8000 apparatus (Shelton, CT, USA). Compounds weighing approximately 10–15 mg were placed in a corundum dish. The measurement was conducted in the temperature range 30–800 °C and under oxidant atmosphere (30 mL/min), at a heating rate of 20 °C/min. The obtained results are the average of three measurements per sample.

The morphology of samples surfaces was observed with a JEOL 5610 scanning electron microscope (Tokyo, Japan). Before observation, the samples were covered with a fine gold–palladium layer in order to increase their conductivity in a vacuum chamber.

The tensile properties of the vulcanized rubber EPDM–EPDMd compounds were assessed following the ISO 37 standard. Testing was conducted using a high-precision Instron 3366 testing machine from the USA, boasting a cell load capacity of 20 kN. The tensile tests were executed at a constant cross-head speed of 500 mm/min, maintaining a controlled environment with a relative humidity (RH) of 50 +/− 5% and a temperature of 23 +/− 2 °C.

For hardness assessments, a Zwick 3130 durometer Shore A from Germany was used, following the ISO 7619-1 standard. To ensure precision and reliability, both tensile and hardness evaluations were based on an average of five measurements per sample. This methodology guarantees the accuracy and consistency of the obtained results, providing a robust foundation for the analysis of the material’s mechanical properties.

The chemical structure of the EPDM-XEPDMd samples was determined by Fourier-Transform Infrared Spectroscopy Attenuated Total Reflectance (FTIR-ATR). The analysis was performed by means of a Spectrum Two spectrometer from Perkin Elmer (USA). The device had an ATR attachment with a diamond crystal. Spectra were registered at 2 cm^−1^ resolution and 40 scans in the range of 500–3500 cm^−1^.

Acoustic characterization was performed using the two-microphone impedance tube method, following the ISO 10534-2 standard with a Brüel & Kjær impedance tube. Cylindrical test specimens, with a thickness of 3 mm and a diameter of 29 mm, were precision-cut from the material plaques and securely positioned within the impedance tube. During the test, a plane sound wave was directed through the sample, and the resulting sound pressure was simultaneously recorded at two distinct microphone positions. The gathered data was routed through an amplifier to specialized software. This software then calculated the sound absorption, determining the relationship between the acoustic energy absorbed by the samples and the total incident acoustic energy. This methodology ensures the accurate and standardized measurement of the material’s sound absorption performance.

## 3. Results

### 3.1. Mechanical Characterization

**Figure 1:**
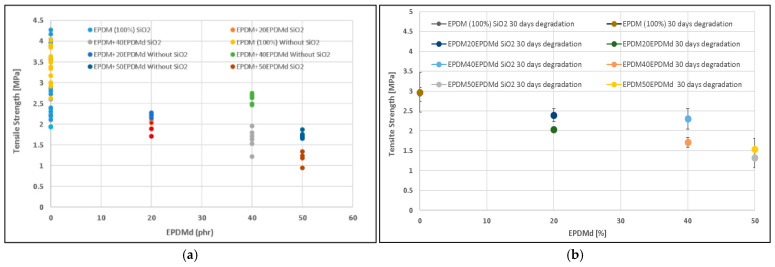
Tensile strength of the (**a**) EPDM–EPDMd compounds with and without EPDMd and (**b**) EPDM–EPDMd compounds with and without EPDMd degraded for 30 days.

Figure 1a shows that the tensile strength (TS) is lower in EPDM compounds reinforced with silica (SiO_2_) compared to those without, suggesting that the SiO_2_ acts primarily as an unfunctionalized filler and introduces stress concentration points rather than effective reinforcement. Furthermore, the incorporation of EPDMd (devulcanized) causes a decrease in TS, which becomes more pronounced above 40 phr, as the EPDMd begins to form aggregates, resulting in a structurally weaker two-phase morphology [18]. This structural heterogeneity also explains the observed increase in the standard deviation in samples containing both SiO_2_ and high EPDMd content, where the SiO_2_ particles act as stress generation centers that initiate microporosity and microcracks, also influenced by the lower interfacial adhesion and reduced interaction degree inherent to the recycled EPDMd component, leading to variable and premature failure.

Figure 1b shows the TS of the EPDM–EPDMd samples degraded over 30 days. The evolution of these samples is similar to that of the same samples without degradation. Considering the standard deviation, thermo-oxidative degradation at 80 °C causes few changes in these samples. The interaction between the EPDM and EPDMd compounds is not affected by the degradation conditions. The TS is acceptable even with 40EPDMd samples without SiO_2_; in degraded samples, the addition of SiO_2_ improves the TS.

**Figure 2:**
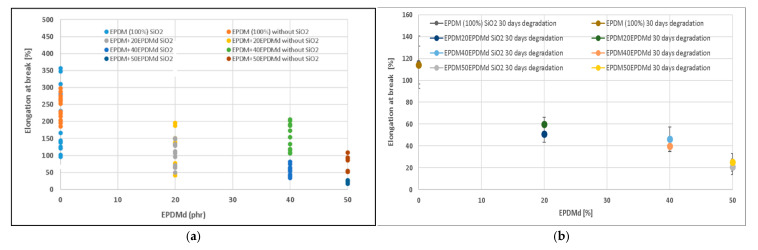
Elongation at break of (**a**) EPDM–EPDMd compounds with and without SiO_2_. (**b**) EPDM–EPDMd compounds degraded for 30 days with and without SiO_2_.

The elongation at break (EB) values of the non-degraded samples, shown in Figure 2a, exhibit a behavioral trend similar to that observed for the tensile strength (TS). For the pure EPDM100 samples, the EB values with and without SiO_2_ are nearly identical, with overlapping standard deviations. This lack of influence from SiO_2_ suggests that, in the neat matrix, the silica particles (with size > 100 microns) are well-distributed and do not act as significant impediments to chain movement or fracture initiators. The initial addition of 20 phr EPDMd causes a clear decrease in EB (approximately 61%), attributable to the reduced chain mobility and inherent inelasticity introduced by the recycled component; however, the EB values for samples with and without SiO_2_ remain within the same range, indicating that the SiO_2_ distribution remains good with the presence of EPDMd. As the EPDMd content increases to 40 phr, the EB values stabilize at the level seen at 20 phr, but a notable divergence emerges; the SiO_2_-containing samples now show significantly lower EB values (approximately 40% less) than their SiO_2_-free counterparts. This difference signifies that at high EPDMd concentrations, the silica particles are no longer perfectly distributed; instead, they preferentially localize within one phase (or at the interface) due to the formation of a differential two-phase structure with the EPDMd, leading to stress concentration and premature failure. This detrimental effect is further magnified at 50EPDMd, where the presence of SiO_2_ causes an even more significant decrease (approximately 30% reduction) in EB compared to the corresponding SiO_2_-free samples, confirming that the SiO_2_ distribution is strongly compromised by high concentrations of the EPDMd phase.

Figure 2b shows the evolution of the EB for the samples degraded for 30 days. A significant decrease in the EB is observed compared to the non-degraded samples, regardless of incorporating SiO_2_ or not (from 250 to 120%). Samples degraded with 20 phr of EPDMd decreased up to 55%, stabilized up to 40% with EPDM40EPDMd samples, and progressed up to 20–25% in EPDM50EPDMd samples. As can be seen, thermo-oxidative degradation significantly affects the EB, although high EPDMd contents also generate a decrease in the EB. However, it should also be noted that the samples with SiO_2_ are more affected than those without SiO_2_.

The evolution of the elongation at break (EB) for the samples subjected to 30 days of thermo-oxidative degradation is presented in Figure 2b. A significant decrease in the EB is observed compared to the non-degraded samples, regardless of incorporating SiO_2_ or not (from 250 to 120%). This loss of elasticity is primarily a consequence of chain scission and cross-linking induced by the thermal oxidation, which severely stiffens the elastomeric matrix. The inclusion of EPDMd compounds exacerbates this effect; samples degraded with 20 phr of EPDMd decrease up to 55%, stabilize up to 40% with EPDM40EPDMd samples, and progress up to 20–25% in EPDM50EPDMd samples. As can be seen, thermo-oxidative degradation significantly affects the EB, although high EPDMd contents also generate a decrease in the EB. However, it should also be noted that the samples with SiO_2_ are more affected than those without SiO_2_.

**Figure 3:**
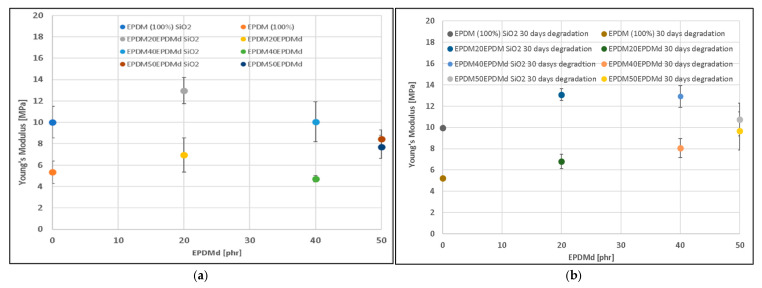
Young’s modulus of (**a**) EPDM–EPDMd compounds with and without SiO_2_. (**b**) EPDM–EPDMd compounds degraded for 30 days with and without SiO_2_.

Figure 3a presents the Young’s modulus (YM) values for the non-degraded EPDM compounds, revealing a dependence on both filler and devulcanized content. The first difference is the effect of the filler; samples containing SiO_2_ exhibit a substantially higher YM (up to two times greater) than their SiO_2_-free counterparts, confirming that the silica successfully acts as a rigid filler and stiffening agent within the EPDM matrix. Conversely, the incorporation of EPDMd causes only a slight, marginal increase in YM at the 20 phr level, with values stabilizing for 40 and 50 phr EPDMd contents. Notably, at these high EPDMd concentrations, the difference in YM between samples with and without SiO_2_ is smaller than in the neat EPDM. This reduction in the stiffening effect of SiO_2_ at high EPDMd loads is attributed to the formation of aggregates of EPDMd within the EPDM matrix, where the EPDMd microaggregates form a stable secondary phase that itself provides a level of inherent reinforcement [17,18], diminishing the relative contribution of the SiO_2_ filler to the overall stiffness. These results are also related to the changes described in the discussion about the elongation at break, so the interplay of the involved phenomena should be considered in order to envision the behavior of the material.

**Figure 4:**
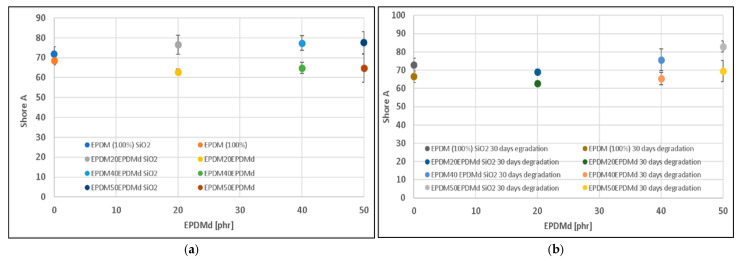
Hardness of (**a**) EPDM–EPDMd compounds with and without SiO_2_. (**b**) EPDM–EPDMd compounds degraded for 30 days with and without SiO_2_.

Thermo-oxidative degradation for 30 days results in a significant increase in both the YM (Figure 3b) and hardness (Figure 4a,b) across all formulations. This widespread stiffening is the expected consequence of oxidative aging, which promotes additional cross-linking and chain entanglement within the EPDM matrix. The SiO_2_-containing samples remain significantly stiffer, maintaining the trend observed in the non-degraded state (Figure 3b). However, the stiffening effect of EPDMd also becomes more prominent; YM slightly increases with 20 phr EPDMd and then stabilizes for 40 and 50 phr EPDMd. As observed in the non-degraded state, the YM values for samples with and without SiO_2_ tend to converge as the EPDMd content increases. This suggests that the EPDMd microaggregates not only act as reinforcement in the original blend but also stabilize the matrix during aging, partially offsetting the stiffening contribution of the SiO_2_ or reaching a plateau of maximum achievable stiffness more quickly due to the complex interaction between the filler and the recycled phase.

### 3.2. Structural Characterization (FTIR-ATR)

**Figure 5:**
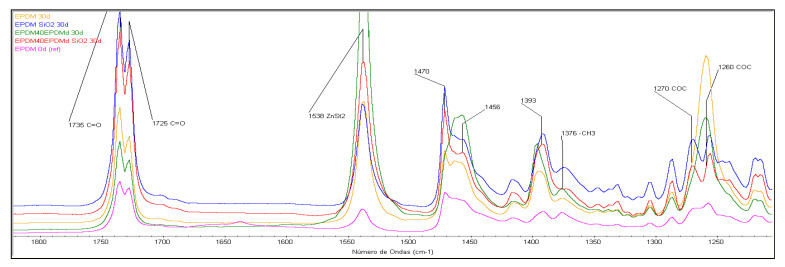
FTIR spectra in the 1800–1200 cm^−1^ region of EPDM and EPDM40EPDMd blends, with and without SiO_2_.

Figure 5 presents the structural characterization of different blends, comparing significant samples containing 15 phr SiO_2_ and 40 phr EPDMd. Following 30 days of thermo-oxidative aging, significant spectral differences are evident. Samples containing SiO_2_ exhibit a higher degree of degradation compared to those without these particles.

**Figure 6:**
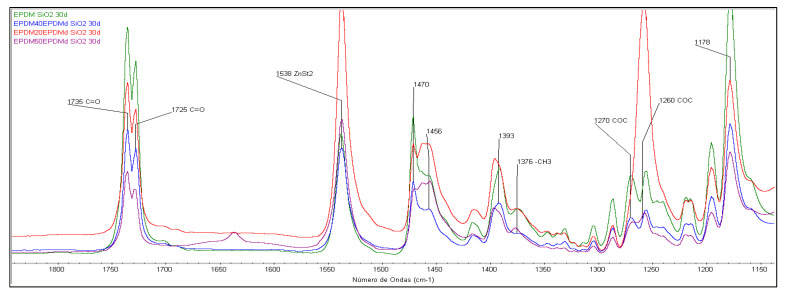
FTIR spectra in the 1850–1100 cm^−1^ region of EPDM and 0, 20, 40, and 50 EPDMd blends, after degradation, with and without SiO_2_. Spectral area of EPDM-XEPDMd with SiO_2_ (X from 0 to 50 phr).

Conversely, as shown in Figure 6, the presence of EPDMd appears to slightly mitigate this oxidative process. The degradation mechanism does not involve the formation of unsaturations, as evidenced by the absence of bands at 1606 cm^−1^. Instead, oxidation is characterized by the emergence of C=O groups in the 1740–1730 cm^−1^ region, a band that shows significantly greater growth in SiO_2_-containing samples. An increase in the 1270–1260 cm^−1^ band, attributed to the formation of C-O-C ether linkages, is also observed [18,19,20]. Finally, the appearance of the characteristic ZnSt_2_ band at 1538 cm^−1^ correlates with EPDMd content, resulting from the exudation of this curing byproduct from the bulk material to the surface.

This phenomenon has been referenced in other publications [17,21]. The spectra in Figure 5 and Figure 6 show how the bands at 1470 and 1456 cm^−1^, assigned to the different methylene groups of the elastomer, present different evolutions depending on the EPDMd content.

EPDM’s excellent stability stems from its largely saturated main chain, devoid of carbon–carbon double bonds or polar groups. Nevertheless, unsaturations present in its diene side chains render it susceptible to oxidation under thermo-oxidative degradation conditions [22]. While EPDM degradation primarily involves crosslinking reactions that do not introduce new IR-active functional groups, the presence of SiO_2_ leads to the formation of characteristic oxygen-containing groups, as previously discussed.

### 3.3. Thermal Characterization (TGA)

According to Ghosh et al. [23], the thermal stability of vulcanized elastomers is highly dependent on their environment, with factors such as main chain configuration, bond energy, crosslinking nature, and interlinks significantly affecting their thermal behavior. Given the influence of these parameters on thermo-oxidative degradation and the presence of EPDMd micro agglomerates within the EPDM matrix, a detailed thermogravimetric analysis (TGA) was conducted. The decomposition temperature at various weight losses (T20, T50, T70), the temperature at maximum weight loss (Tmax), the rate of weight change at Tmax, and the inorganic residue are presented in Table 1.

In non-degraded samples, the presence of SiO_2_ generally leads to higher decomposition temperatures (TdT), stabilizing the material by restricting the mobility of elastomeric chains [24,25], acting as a thermal barrier, or protecting the macromolecular structure of EPDM [26,27], provided its dispersion within the EPDM matrix is adequate. For example, SiO_2_ incorporation increased the TdT from 465 °C to 471 °C in 100EPDM samples and from 455 °C to 465 °C in EPDM50EPDMd samples. Conversely, the incorporation of EPDMd, which generates dispersed microagglomerates with a lower crosslinking density [19], tends to decrease the TdT of the compound. Consequently, in EPDM–EPDMd/SiO_2_ composites, the beneficial effect of SiO_2_ on TdT is diminished with increasing EPDMd content. Then, in EPDM–EPDMd SiO_2_ compounds, the effect of SiO_2_ is lower and there is a decrease in the TdT when the EPDMd content is higher.

Furthermore, the analysis of Table 1 also reveals that 30 days of thermo-oxidative degradation significantly impact the TGA profiles, primarily through the evaporation of volatile components. The high plasticizer content within EPDMd notably decreases the T20 as EPDMd content increases. This volatilization generates micropores, making the EPDMd phase more vulnerable to thermal decomposition, although it may favor the acoustic properties in degraded samples. However, it is also observed that in degraded samples, SiO_2_ causes a decrease in TdT (an effect opposite to that observed in non-degraded samples), thus acting as a catalyst for the thermodegradative process. This behavior, corroborated by FTIR-ATR analysis and other studies [28], is attributed to two main mechanisms. (a) Change in polymer chain dynamics: The presence of silica can alter the movement and relaxation of polymer chains (due to proximity, mobility restriction, etc.), making certain reactions (chain scission, local oxidations) more likely at points of stress or at interfaces [29]. (b) Adsorption of oxygen on the SiO_2_ surface: This adsorption facilitates the generation of reactive species, such as peroxides, which then act upon the elastomeric chain [30].

### 3.4. Acoustic Characterization

**Figure 7:**
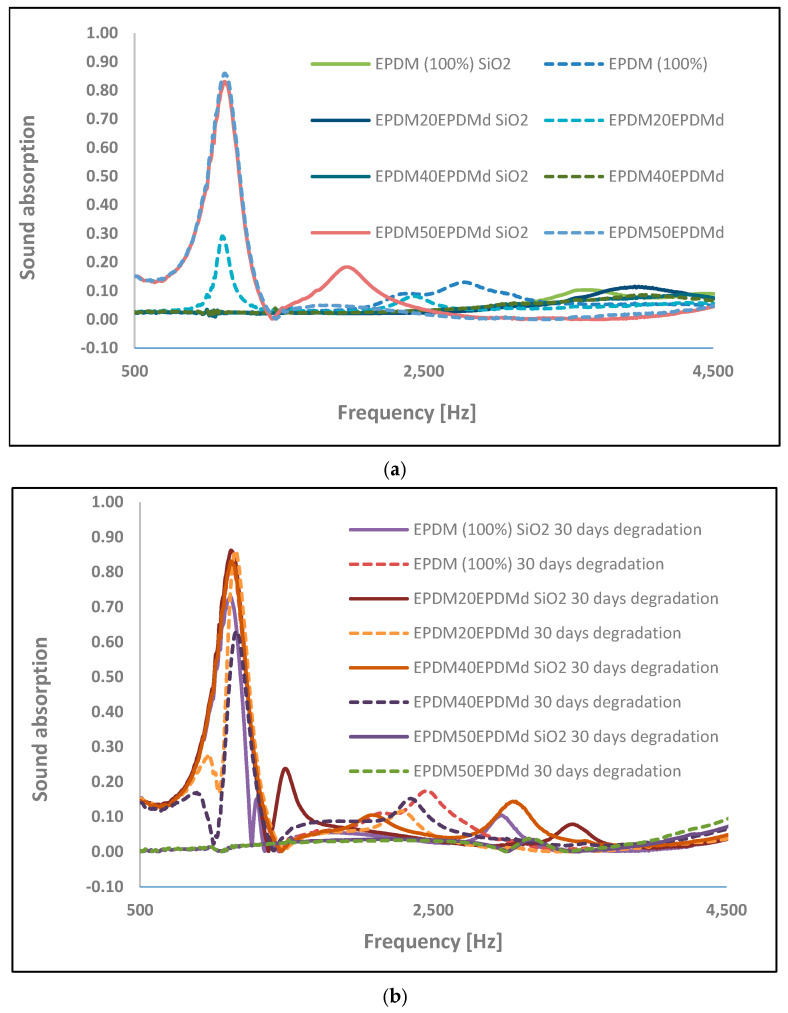
Sound absorption of EPDM/EPDMd samples: (**a**) non-degraded samples; (**b**) degraded samples.

As can be seen in Figure 7a,b, the sound absorption of the EPDM–EPDMd samples with and without SiO_2_, depending on the EPDMd content, is quite poor and only absorbs at low frequencies (<1500 Hz). This behavior indicates that, despite the elastomeric nature of the material, the surface acoustic impedance still exhibits a certain mismatch with the air, thereby limiting the access of high-frequency sound waves.

However, at low frequencies, the samples degraded for 30 days display better acoustic behavior than the samples without degradation. The significant difference between the 50EPDMd sample without degradation compared to the other samples is due to the fact that the microaggregates generated by the EPDMd cause more microcavities, which improve absorption at low frequencies [31,32,33]. This structure increases the effective porosity ratio of the material, creating new pathways for energy dissipation. However, the thermo-oxidative degradation process at 80 °C allows the release of volatiles from the EPDMd microaggregates, also favoring the formation of microcavities that allow better acoustic absorption [34,35]. This release of volatiles, mainly EPDMd plasticizer, is observed in the T_20_ values in Table 1. This volatilization not only increases the porosity ratio by leaving additional voids but also modifies the internal pore distribution, generating a network of interconnected cavities that favors the viscous dissipation of long-wavelength sound waves (low frequencies). This increase in porosity also contributes to reducing the apparent density of the material, providing better acoustic impedance and allowing greater diffusion of sound energy into the matrix.

### 3.5. SEM Microstructural Analysis

**Figure 8:**
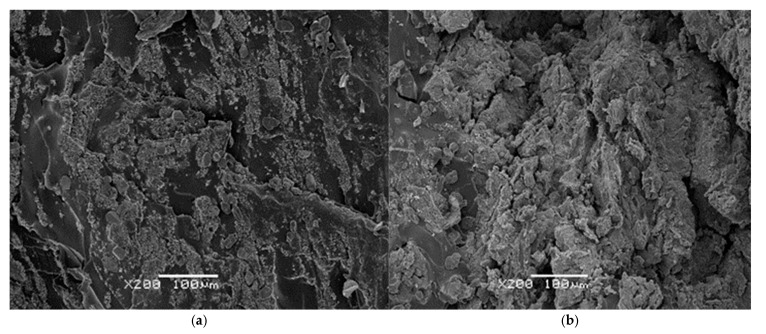
SEM micrographs of EPDM100 samples with SiO_2_: (**a**) non-degraded and (**b**) degraded for 30 days. Magnification: ×200.

In Figure 8a,b, SiO_2_ particles and surface-adsorbed ZnSt_2_ are discernible, predominantly in the non-degraded sample. The degraded sample, however, displays a markedly more irregular surface, featuring numerous microcavities and cracks attributed, as commented previously, to the volatilization of low molecular weight compounds.

**Figure 9:**
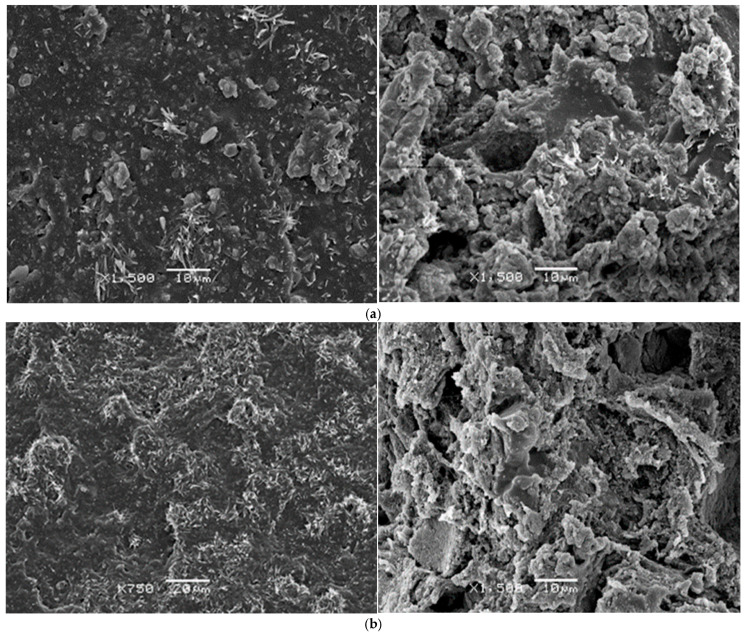
SEM micrographs of the samples (**a**) EPDM20EPDMd SiO_2_ without degradation and degraded for 30 days (magnification 1500×) and (**b**) EPDM50EPDMd SiO_2_ without degradation and degraded for 30 days (magnifications 750× and 1500×).

Figure 9 presents micrographs of both initial and degraded EPDM20EPDMd/SiO_2_ and EPDM50EPDMd/SiO_2_ samples. These images show the EPDMd microaggregates, which retain the crystallized ZnSt_2_ particles. As the EPDMd content increases, a greater number of microaggregates are generated, leading to a higher accumulation of ZnSt_2_ crystals within these structures. Following degradation, the samples exhibit a significantly more irregular structure, characterized by cracked microcavities. In these degraded regions, the ZnSt_2_ crystals become less discernible, suggesting that the EPDM microaggregates have dispersed and consequently lost their ability to agglutinate the ZnSt_2_ crystals.

These microstructural observations provide strong corroboration for the results obtained from mechanical, thermal, and FTIR-ATR analyses. Collectively, they demonstrate that the presence of SiO_2_ catalyzes the thermo-oxidative degradation process in the EPDM elastomeric samples.

## 4. Discussion

After analyzing the results, it can be considered that the elastomeric materials EPDM/EPDMd can be used in the construction and building area for the following reasons.

In mechanical and structural behavior, the addition of silica is clearly advantageous since it increases the Young’s modulus (stiffness), compensating for the slight loss of tensile strength that recycled rubber can provoke. This is ideal for structural components or joints that require dimensional stability.

In terms of acoustic properties, the results obtained are very interesting because the incorporation of recycled EPDM and silica does not reduce acoustic absorption and, in low frequency ranges, improves it. This makes the material technically suitable for insulating panels.

Although degradation increases hardness and stiffness (due to post-crosslinking), the material maintains its integrity and offers acceptable thermal stability for use in construction.

In the part that refers to sustainability benefits and functional trade-offs, the study suggests additional benefits where silica not only reinforces, but also acts as a barrier (TGA) that modulates degradation. A material that maintains its properties for longer reduces the need for maintenance and replacement in buildings, improving the life cycle (LCA). However, in the circular economy, the use of dEPDM plays an important role in that it allows the circle to be closed, preventing this waste from ending up in landfill or incinerators, transforming waste into a raw material of high technical value.

The study also shows that with a higher silica content, the rigidity increases but the elongation decreases (the material becomes more fragile). In construction and building, this implies that for very flexible membranes, the % of silica should be limited, while for vibration supports or rigid panels, the increase in silica is a significant benefit.

## 5. Conclusions

This study investigated the properties of EPDM and devulcanized EPDM (EPDMd) composites at varying ratios, with and without SiO_2_, under thermo-oxidative degradation. Regarding mechanical properties, SiO_2_ incorporation significantly enhanced Young’s modulus (e.g., from 7 to 13 MPa for 20EPDMd samples) while largely maintaining tensile strength (approx. 2 MPa for 20EPDMd samples), though it led to a notable decrease in elongation at break (e.g., from 150% to 60% for 40EPDMd samples). Conversely, EPDMd incorporation slightly reduced both tensile strength (3.3 MPa for EPDM100 to 2 MPa for 20EPDMd) and elongation at break, but increased Young’s modulus (e.g., 10 MPa for EPDM100/SiO_2_ to 13 MPa for 20EPDMd/SiO_2_).

Thermo-oxidative degradation (30 days) did not significantly affect tensile strength, but it increased Young’s modulus for samples with EPDMd content > 40 phr (both with and without SiO_2_) and significantly decreased elongation at break across all samples and EPDMd contents (e.g., 250% to 115% in EPDM100 samples). Comprehensive analyses by FTIR-ATR, TGA, and SEM confirmed that SiO_2_ catalyzes the thermo-oxidative degradation process, whereas EPDMd conversely inhibits the structural degradation of these elastomers. It was also observed that EPDMd forms microaggregates that effectively retain exuded ZnSt_2_ crystals, especially in non-degraded samples. The degraded samples developed highly irregular structures, characterized by the presence of microcavities and cracks, generating occlusions. Crucially, these degradation-induced occlusions significantly enhanced the acoustic absorption properties of the degraded samples, particularly at frequencies below 1500 Hz.

The mechanism proposed for using SiO_2_ as a catalyst is based on the reactivity of the surface Silanol groups present in the particles, which are covered with hydroxyl groups, which can generate acidity that, under conditions of thermal stress, can catalyze chemical reactions in elastomeric materials [36,37].

In summary, the study validates the suggestion that the use of recycled rubber is not only an “ecological” option but a robust technical solution that meets the standards required in the modern construction sector.

## Figures and Tables

**Table 1:** Degradation temperatures of different EPDM/EPDMd samples.

Sample Code	Degradation Temperature (°C)			T_max_ (°C)	Weight Loss Rate at T_max_ (%/s)	Residue (%)
	T_20_	T_50_	T_70_			
**EPDM100**	424.6	467.1	494.3	467.1	0.00452	2.20
**EPDM20EPDMd**	432.5	468.9	496.2	472.6	0.00472	2.23
**EPDM40EPDMd**	422.0	468.3	523.1	470.3	0.00325	3.01
**EPDM50EPDMd**	419.1	469.2	566.2	469.0	0.00310	7.15
**EPDM100 SiO_2_**	387.9	470.8	576.4	471.3	0.00373	11.03
**EPDM20EPDMd SiO_2_**	416.4	475.6	616.8	475.2	0.00329	10.58
**EPDM40EPDMd SiO_2_**	386.0	462.3	518.6	478.0	0.00429	17.77
**EPDM50EPDMd SiO_2_**	381.0	491.1	627.7	480.5	0.00248	23.94
**EPDM100 30 days**	403.8	467.1	494.3	465.1	0.00464	2.17
**EPDM20EPDMd 30 days**	399.5	468.9	496.2	462.2	0.00479	2.13
**EPDM40EPDMd 30 days**	380.0	472.3	523.1	487.6	0.00344	2.91
**EPDM50EPDMd 30 days**	376.3	489.2	566.2	485.4	0.00318	7.02
**EPDM100 SiO_2_ 30 days**	407.1	460.8	568.4	461.9	0.00379	10.63
**EPDM20EPDMd SiO_2_ 30 days**	400.3	465.6	596.8	462.2	0.00343	10.98
**EPDM40EPDMd SiO_2_ 30 days**	376.0	462.3	598.0	475.0	0.00406	16.37
**EPDM50EPDMd SiO_2_ 30 days**	379.4	471.6	587.1	477.5	0.00237	21.91

## Data Availability

The original contributions presented in this study are included in the article. Further inquiries can be directed to the corresponding authors.
